# Annotations

**Published:** 1911-06-17

**Authors:** 


### Annotations.

### Hygienic Risks of the King's Fete to Children.

The assembling of a hundred thousand school children at the Crystal Palace on June 30 is an event of public importance, not only from the imperial point of view but also from the hygienic. To convey so large a party is in itself a stupendous task, and we may sympathise with the organisers and the teachers who will be in charge of this tremendous Coronation celebration. In the interests of public health it is well to bear in mind the practical certainty of a notable increase in cases of infectious disease after such a congregation of children. Consideration must be given to the fact that among such a large number a certain percentage of children will be in the incubation stage of some disease or other, and at such times the eagerness to be included among the favoured ones is apt to lead to the suppression of information. A fête such as this, preceded as it will be by a journey in closely-packed railway carriages, is of course eminently favourable to the dissemination of the specific fevers. It is to be hoped that during the week preceding the event as far as possible the recent infectious history of the selected families will be ascertained. In the ordinary course of school work the attendance officers are kept pretty well posted in this matter, and with a little extra care it should be possible to avoid an appreciable amount of the inevitable risk. The actual fatigue to be endured by the children in the course of the day is enough to inflict considerable strain on the healthiest, and we are glad to see that an efficient nursing and ambulance service is to be available on that occasion.

### The Modern Pheidippides.

Those who have seen the competitors in a so-called "Marathon" race struggling home, haggard, exhausted, and agonised, might wonder if the prize offered can be any compensation for the suffering endured. We do not speak of its pecuniary value, which is not a very great temptation, but even the personal and national prestige that may be gained. It is evident that some of the competitors are strained up to, and even beyond, the fair limit of their powers. We presume that none enters on such a race without a medical examination that will assure him that he suffers no immediate danger from taking part in it, but one would fain be assured that the limp, wretched figures whom one sees are not men who will, in the future, without incurring equal fatigue, suffer as much from aching muscles, gasping lungs, and overstrained heart as they seem to do at the moment when their effort ends. One would like to have an actuarial estimate of the expectation of life of, say, Dorando—as he is perhaps the best known of these Marathon runners—before his great feat and as it stands to-day. Has he or has he not suffered from the strain? Could he, any time within the next ten years, repeat it? Is he a stronger man or a weaker than he was before he made the attempt? These are questions which are worth considering—worth answering. For, although it may be permissible and honourable for a man to exert himself to his utmost limit, and even to die in some great task in the time of his country's need, it does not follow that it is worth while to let men exhaust their strength in a feat which gives only a day's excitement. Reasonable and progressive athletic efforts are good, but it is doubtful if it is wise to strain a number of men to the utmost limit ever known to have been attained by any mortal. Pheidippides, the swift runner, was the only one in Attica who could have achieved the feat of running from Marathon to Athens, while we expect many to be able to emulate it. He made his great effort in a great cause, while we permit men to do it for a mere trivial triumph. And, having achieved his aim, Pheidippides fell down dead. Will it not be time to use and kill our Pheidippides when the cry has to be uttered, "Athens is saved"?

### The Unpopularity of Cremation.

Though the practice of cremation seems destined to increase extensively both from its hygienic advantages and from the continually unsatisfied demands in every urban centre for new places of burial, it is surprising to find from Dr. Collingridge's report, as Medical Officer of Health for the City, that in the United Kingdom last year there were only 840 cremations. Contrast this with the number of deaths in London alone, which he gives as 72,000 a year, and we are compelled to admit the conservatism of our national prejudices. It is supposed that cremation is expensive, but we learn that the Ilford Corporation will perform it for £2 15s. 6d. Woking was the first place to construct a crematorium in 1885, and now, though Manchester, Glasgow, Liverpool, Hull, Darlington, Leicester, Golder's Green, Birmingham, Bradford, Leeds, Sheffield, and Ilford have followed its example, in the twenty-five years that have passed only 8,960 cremations have taken place. Medical men must wish that Royal obsequies could set a public example, and perform an invaluable public service, by including this practice.

### The Nastin Treatment for Leprosy.

A valuable report on the Nastin Treatment for Leprosy at the Mahaica Leper Asylum, British Guiana, has been published as a Parliamentary Paper (Cd. 5583). Dr. R. S. Wise, the Government bacteriologist, who is the author of the report, expresses himself cautiously, but it is stated that since the Nastin treatment has been introduced four lepers have been discharged as cured, and at the present time there are fifty-six cases in which no leprosy bacilli can be found. In the opinion of Dr. Wise, however, the favourable results are so few that longer and wider experience can alone determine whether the favourable cases were instances of natural improvement or whether the injections of Nastin played an active part in the disappearance of the leprous deposits. The action of the injections of Nastin appears to be to initiate, accelerate, or intensify a natural process which usually occurs in too small a quantity or too late in the disease to benefit the patient.

